# Synergistic Use of Geniposide and Ginsenoside Rg1 Balance Microglial TNF-**α** and TGF-**β**1 following Oxygen-Glucose Deprivation* In Vitro*: A Genome-Wide Survey

**DOI:** 10.1155/2015/756346

**Published:** 2015-11-29

**Authors:** Jun Wang, Jincai Hou, Hui Zhao, Jianxun Liu

**Affiliations:** ^1^Institute of Basic Theory, China Academy of Chinese Medical Sciences, 16 Dong Zhi Men Nei Nan Xiao Jie, Dongcheng District, Beijing 100700, China; ^2^Xiyuan Hospital of China Academy of Chinese Medical Sciences, 1 Xiyuan Caochang, Haidian District, Beijing 100091, China; ^3^China Academy of Chinese Medical Sciences, 16 Dong Zhi Men Nei Nan Xiao Jie, Dongcheng District, Beijing 100700, China

## Abstract

Ischemia-activated microglia are like a double-edged sword, characterized by both neurotoxic and neuroprotective effects. The aim of this study was to reveal the synergistic effect of geniposide and ginsenoside Rg1 based on tumor necrosis factor- (TNF-) *α* and transforming growth factor- (TGF-) *β*1 balance of microglia. BV2 microglial cells were divided into 5 groups: control, model (oxygen-glucose deprivation (OGD)), geniposide-treated, ginsenoside-Rg1-treated, and combination-treated. A series of assays were used to detect on (i) cell viability; (ii) NO content; (iii) expression (content) of TNF-*α* and TGF-*β*1; and (iv) gene expression profiles. The results showed that integrated use of geniposide and ginsenoside Rg1 significantly inhibited NO level and protected cell viability, improved the content and expression of TGF-*β*1, and reduced the content and expression of TNF-*α*. Separated use of geniposide or ginsenoside Rg1 showed different effects at different emphases. Next-generation sequencing showed that Fc*γ*-receptor-mediated phagocytosis pathway played a key regulatory role in the balance of TNF-*α* and TGF-*β*1 when cotreated with geniposide and ginsenoside Rg1. These findings suggest that synergistic drug combination of geniposide and ginsenoside Rg1 in the treatment of stroke is a feasible avenue for the application.

## 1. Introduction

Stroke is a leading cause of morbidity and mortality in humans and results from occlusion or hemorrhage of blood vessels. Emerging data from preclinical studies [1, 2] and randomized control trials [3, 4] demonstrate that combination therapy provides a survival advantage and increases the treatment effect for ischemic stroke without substantially increasing the side effects. Millennia-old traditional Chinese medicine (TCM) is widely used in clinical treatments for stroke through combination therapies involving multiple ingredients. However, the complex combination of ingredients makes it difficult to determine the mechanism of interaction among the ingredients for treating ischemic stroke [5].

Over several decades, the question of whether microglia play harmful or beneficial roles in CNS injury has been widely debated and reviewed [6–8]. Microglia have been confirmed to possess neurotoxic effects after transient cerebral ischemia [9] by releasing tumor necrosis factor- (TNF-) *α*, interleukin- (IL-) 1*β*, IL-6, NO, and reactive oxygen species (ROS) [10, 11]. Activated microglia have also been reported to possess neuroprotective/neurotrophic effects* in vitro* and* in vivo*. Under certain conditions, microglial cells are able to produce anti-inflammatory cytokines such as IL-10 and transforming growth factor- (TGF-) *β*, which have neuroprotective effects in experimental animal models of traumatic injury and stroke [7, 8]. The exogenous microglia directly applied to the hippocampal slice cultures could save more neurons after deprivation of oxygen and glucose [12]. In brief, microglial cells are able to produce and release a plethora of soluble mediators ranging from cytotoxic mediators to trophic factors, which can exert deleterious as well as beneficial effects on the surrounding tissue [13].

Damage to the CNS leads to the increased production of TNF-*α* and TGF-*β*1 cytokines that have pro- or anti-inflammatory actions, respectively [14]. Microglial cells are the major source of TNF-*α* and TGF-*β*1. The effects of geniposide on microglial cells mainly focus on the regulation of TNF-*α* [15, 16], while the effects of ginsenoside Rg1 mainly focus on the regulation of TGF-*β*1 and were usually used in antifibrotic therapy [17]. Despite the reports of monotherapy of geniposide or ginsenoside Rg1, seldom study of geniposide and ginsenoside Rg1 synergy was performed. Geniposide and ginsenoside Rg1 (Figure S1 in Supplementary Material available online at http://dx.doi.org/10.1155/2015/756346) are bioactive compounds that are, respectively, extracted from Cape Jasmine Fruit (*Fructus Gardeniae*) and Sanchi (*Radix Notoginseng*) [18], two Chinese couplet medicines that have been used for the treatment of stroke in China. We have demonstrated that the combination of geniposide and ginsenoside Rg1 (prescribed as Tongluo Jiunao injection) can reduce expression of macrophage inflammatory protein- (MIP-) 1*β* and chemokine CC receptor 5 in oxygen-glucose deprivation- (OGD-) injured microglial cells, as well as inhibit the proliferative activity of microglial cells, suggesting the therapeutic potential of the combination of geniposide and ginsenoside Rg1 on ischemic cerebral vascular disease [19]. In addition, our previous work showed that geniposide inhibited the activation of microglial cells in ischemic brain tissue of the rat injured middle cerebral occlusion (MCAO)* in vivo*. Meanwhile,* in vitro* cultured microglia were also used to demonstrate that the level of TNF-*α* secreted by microglia was also suppressed by geniposide [20]. Our endpoint of this presented study was to understand whether synergistic use of geniposide and ginsenoside Rg1 can integrate microglial TNF-*α* and TGF-*β*1 pathways and balance microglial TNF-*α* and TGF-*β*1.

## 2. Materials and Methods 

### 2.1. BV2 Microglial Cell Culture

The murine BV2 microglial cells were grown in T-25 tissue culture cell flasks in 5% CO_2_ and 37°C humidified atmosphere using Dulbecco's Modified Eagle's Medium (DMEM)/F12 culture media supplemented with 10% fetal bovine serum, 2 mM glutamine, and 100 *μ*g/mL penicillin-streptomycin. The microglial cells were maintained via 2 to 3 passages each week.

### 2.2. Establishment of* In Vitro* Hypoxia Model and Drug Administration

Microglial cells were challenged by OGD, as described by our former works [20]. The cell suspension was seeded onto culture plates at a density of 1 × 10^6^/mL. The normal cultured microglial cells without any treatment were used as control. For the microglial cells in OGD, culture medium was changed to glucose-free DMEM, and the culture flasks (or plates) were placed into a sealed tank with a persistent low flow (1.5 L/min) of 95% N_2_ and 5% CO_2_ mixture to expire the oxygen for 20 min. The inlet and outlet ends of the tubes were then clipped, and the tank was placed into an incubator for 6 h to mimic OGD.

The drug preparation used was a chemically standardized product obtained from the National Institutes for Food and Drug Control, which was validated by fingerprint chromatographic methodologies. Microglial cells were divided into 5 groups: control, model (OGD), geniposide-treated (25 *μ*g/mL, geniposide monotherapy), ginsenoside-Rg1-treated (5 *μ*g/mL, ginsenoside Rg1 monotherapy), and combination-treated (geniposide and ginsenoside Rg1, 1 : 1). Microglial cells were preconditioned with geniposide, ginsenoside Rg1, or combination, respectively, for 2 h and then maintained for a further 6 h for hypoxia.

### 2.3. CCK-8 Assay for the Cell Viability

Microglia at 1 × 10^3^ cells per well were seeded on 96-well plates. At the end of OGD, fluids in 96-well culture plates were then changed to DMEM/F12 to avoid background interference and 10 *μ*L of Cell Counting Kit-8 (CCK-8) was added to each well. Each well was measured using a microplate reader with a test wavelength of 450 nm (620 nm as reference wavelength).

### 2.4. NO Assay for the NO Level

At the terminal of OGD, fifty microliters of standard or culture medium from each group was added in each well of 96-well plate, and Griess Reagents I and II were added to the wells. The reaction system was incubated in the dark at room temperature. Ninety-six-well plates were measured with a test wavelength of 540 nm. The NO content of each group was calculated by standard curve.

### 2.5. ELISA Assay

After OGD of 6 h, TNF-*α* and TGF-*β*1 contents were immediately detected by ELISA. Groups and treatments of microglial cells were the same as above. ELISA steps were performed according to the protocol provided by ELISA kit. The TNF-*α* and TGF-*β*1 concentrations of each group were calculated by standard curve. The experiment was repeated in triplicate and six wells were used for each group.

### 2.6. Western Blot Analysis

At the end of OGD, western blot analysis was performed to quantify TNF-*α* and TGF-*β*1 expression in microglia, as well as the validation for the upregulated differential genes according to standard protocols. Microglial cells were washed with ice-cold PBS and scraped in lysis buffer (50 mM Tris and 150 mM NaCl, pH 7.4, containing 1% Triton X-100, 1% Nonidet P-40, 0.5% sodium deoxycholate, 0.1% SDS, 1 mM phenylmethylsulfonyl fluoride, 15 *μ*g/mL leupeptin, 71 *μ*g/mL phenanthroline, and 20 U/mL aprotinin). The insoluble material was removed by centrifugation at 12,000 ×g for 20 min. The protein content was measured according to the bicinchoninic acid method. Twenty micrograms of protein was processed using 12.5% SDS-PAGE and transferred to a 0.45 *μ*m nitrocellulose membrane. Nonspecific binding sites were blocked with TBS (40 mM Tris, pH 7.6, and 300 mM NaCl) that contained 5% nonfat dry milk for 1 h at 37°C. The membrane was then incubated with antibodies against TNF-*α*, TGF-*β*1 (1 : 1000), USP17L (1 : 500), and Fcrlb (1 : 500) and then in a 1 : 5000 dilution of horseradish-peroxidase-conjugated goat anti-rabbit IgG or horseradish-peroxidase-conjugated rabbit anti-goat IgG. Immunoreactive proteins were detected by enhanced chemiluminescence. The experiment was repeated in triplicate and three wells were used for each group.

### 2.7. Immunofluorescence Analysis

The microglial cells were fixed with 4% formaldehyde for 10 min at room temperature and incubated with 0.1% Triton X-100 for 10 min at room temperature. 3% H_2_O_2_ was used to inactivate endogenous peroxidase and 5% BSA was used to block nonspecific binding. The cells were incubated overnight at 4°C with primary antibodies against TNF-*α* (1 : 200), followed by incubation with FITC-conjugated secondary antibody (1 : 200). After the staining procedure, the cells on the coverslips were mounted with triglyceride and visualized with a fluorescence microscope.

### 2.8. Digital Gene Expression

At the terminal of OGD, total cellular RNAs were immediately extracted from the isolated microglia using TRIzol reagent. Ten-microgram RNA samples were amplified with poly-T oligo attached magnetic beads (Invitrogen) according to the manufacturer's instructions. In order to construct the RNA library and sequence, equal quantities of RNA samples from five groups were pooled. Approximately, 5 *μ*g RNA representing each group was submitted to Solexa (now Illumina, San Diego, CA, USA) for sequencing. Sequence tag preparation was done with mRNA-Seq sample preparation kit (Illumina) according to the manufacturer's protocol. High-quality reads were aligned to the mouse reference genome (NCBI Build 36.1) using Bowtie software, which is an ultrafast memory-efficient short read aligner. To compare the different expression of genes across samples, the number of raw clean tags in each library was normalized to tags per million (TPM) to obtain normalized gene expression level. Genes were deemed significantly differentially expressed with a *p* value of 0.005, a false discovery rate (FDR) of 0.01, and an estimated absolute log-2-fold change of 0.5 in sequence counts across libraries.

### 2.9. Real-Time (RT)-PCR

Five nanograms of total RNA was reverse-transcribed using oligo d(T) and Primer Script RT Enzyme MixI (TaKaRa) according to the manufacturer's instructions. cDNA was subjected to real-time quantitative PCR with defined primers and Power SYBR Green PCR Master Mix (Applied Biosystems, Foster City, CA, USA) using the ABI 7000 sequence detection system (Applied Biosystems). The data were analyzed using the ABI 7000 system SDS software. All the experiments were performed in duplicate and relative expression levels of these mRNAs were determined by the 2^−ΔΔCt^ method.

### 2.10. Cluster Analysis

Normalized signal intensities from each experimental condition for the differentially expressed genes were uploaded into Cluster 3.0 software. After log-2 transformation of the data, genes and arrays were median centered, and the resulting data were hierarchically clustered. Gene ratio was expressed by TreeView and output by different color: red representing upregulation and green representing downregulation.

### 2.11. KEGG Analysis

Normalized signal intensities from each experimental condition for the differentially expressed genes were uploaded to the KEGG database, collecting pathway maps that computerize the network information of molecular interaction. KEGG analysis is used for discovering relations that are not easily visible from the changes in individual genes. Pathways that had significant changes of *p* < 0.05 and fold change >1.5 were identified for further analysis.

## 3. Results

### 3.1. Combination of Geniposide and Ginsenoside Rg1 Improved Cell Viability and Reduced NO Release in OGD-Induced Microglia

**Figure 1:**
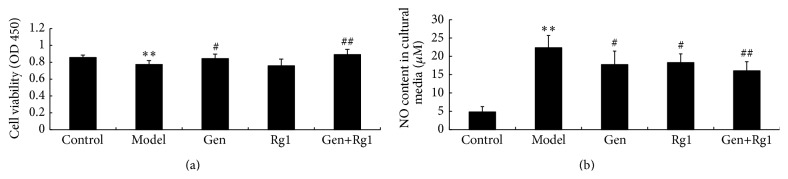
(a) Bar graphs show the changes in microglial cell viability determined by CCK-8 assay. A significant decrease in cell viability was observed when microglia were exposed to hypoxia, and viability increased when microglia were cultured with geniposide. Cell viability increased further when microglial cells were treated with the combination. (b) Bar graphs show the changes in NO content in microglial cultural medium. NO content in the model group was significantly increased compared with the control group. When treated with geniposide or ginsenoside Rg1 alone, the NO content was obviously suppressed. The NO content was suppressed further when microglia were treated with the combination. ^*∗∗*^ *p* < 0.01 versus control, ^#^ *p* < 0.05 versus model, and ^##^ *p* < 0.01 versus model. Control, normal cultured microglial cells; Model, OGD-injured microglial cells; Gen, geniposide-treated microglial cells; Rg1, ginsenoside-Rg1-treated microglial cells; Gen+Rg1, combination-treated microglial cells.

Compared with the control group, the viability of microglial cells in the model group was reduced significantly by OGD injury (Figure 1(a), *p* < 0.01). Reversely, cell viability displayed an obvious improvement after the combinated treatment, which was prior to ginsenoside Rg1 or geniposide monotherapy. NO content in the culture medium in the model group was increased significantly by OGD injury (Figure 1(b), *p* < 0.01) as compared with the control group. Compared with the model group, there was a marked reduction in NO release after combination treatment Rg1 (*p* < 0.01), which was prior to geniposide or ginsenoside Rg1 monotherapy (*p* < 0.05).

### 3.2. Combination of Geniposide and Ginsenoside Rg1 Improved TGF-*β*1 Expression and Reduced TNF-*α* Expression on Microglia and the Contents in Cultured Media

**Figure 2:**
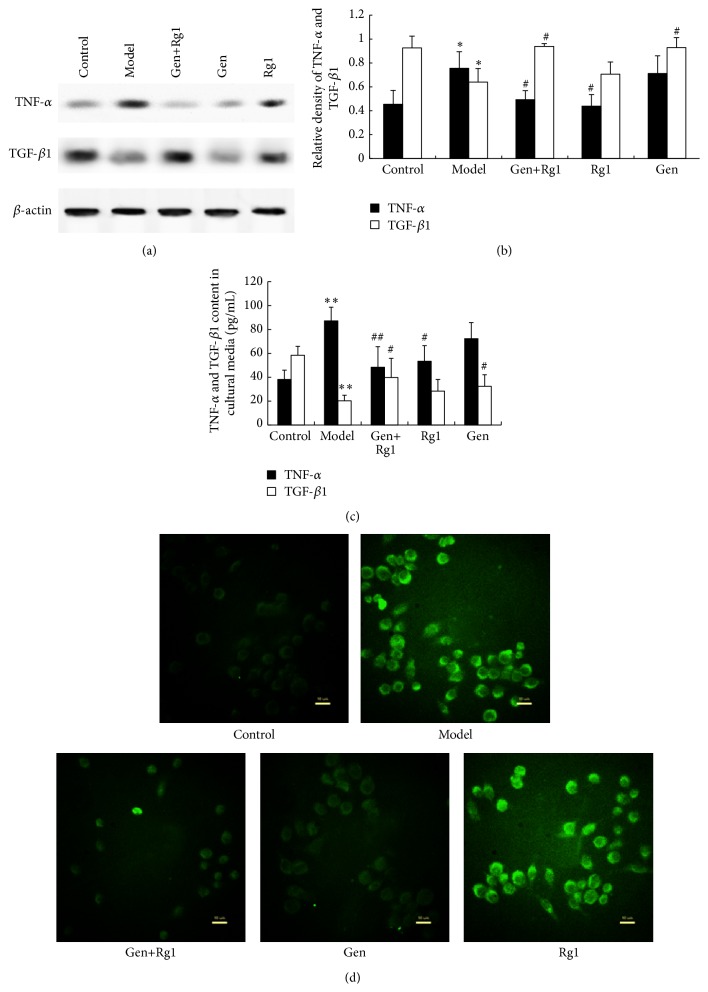
(a) Expression of TNF-*α* and TGF-*β*1 in microglia was assessed by western blotting. Photographs show representative blots of TNF-*α*, TGF-*β*1, and *β*-actin (loading control). (b) Bar graphs show the relative densities of TNF-*α* and TGF-*β*1 bands on western blotting estimated quantitatively by Phoretix 1D image software. Values represent the mean optical density ratio relative to the loading control. (c) Bar graphs show the contents of TNF-*α* and TGF-*β*1 in the cultured media. (d) Immunofluorescence for TNF-*α* in different treated groups. ^*∗*^ *p* < 0.05 versus control, ^*∗∗*^ *p* < 0.01 versus control, ^#^ *p* < 0.05 versus model, and ^##^ *p* < 0.01 versus model. Control, normal cultured microglial cells; Model, OGD-injured microglial cells; Gen, geniposide-treated microglial cells; Rg1, ginsenoside-Rg1-treated microglial cells; Gen+Rg1, combination-treated microglial cells.

As shown in Figure 2, the expression from western blot and content in media from ELISA of TNF-*α* in the OGD group increased by 1.67-fold (*p* < 0.05) and 3.37-fold (*p* < 0.01), respectively, relative to the control. Meanwhile, OGD caused TGF-*β*1 suppression from western blot by 30.96% (*p* < 0.05) and content inhibition in media from ELISA by 68.05% (*p* < 0.01). Compared with the OGD group, ginsenoside Rg1 monotherapy had no effect on TNF-*α* expression and content, which were markedly reduced in the culture media after combinated treatment (*p* < 0.01), with a prior geniposide or ginsenoside Rg1 monotherapy (*p* < 0.05). The effect of combinated treatment on TNF-*α* expression was the same as geniposide monotherapy, with an obvious reduction. The protein level and expression of TGF-*β*1 in the geniposide group were unaffected relative to the model group. However, ginsenoside Rg1 monotherapy increased TGF-*β*1 expression and content in media significantly (*p* < 0.05), with the same effect as combinated treatment. In addition, immunofluorescence labeling of TNF-*α* under different conditions was also carried out to confirm the western blot results.  Figure 2(d) showed that immunofluorescence using TNF-*α* antibody displayed an increase in immunoreactivity in the BV-2 cells of group model compared with the control. The immunoreactivities of geniposide monotherapy and combinated treatment were weaker than group model, with the same tendency as the result of western blot.

### 3.3. Differentially Expressed Genes

136 differentially expressed genes were found in geniposide monotherapy group as compared with the model group, with 54 (39.7%) upregulated and 82 (60.3%) downregulated genes. There were 1383 differentially expressed genes identified in the ginsenoside Rg1 treatment group, with 633 (45.8%) upregulated and 750 (54.2%) downregulated genes. We also determined 430 differentially expressed genes in combination-treated group, with 294 (68.4%) upregulated and 136 (31.6%) downregulated genes.

**Figure 3:**
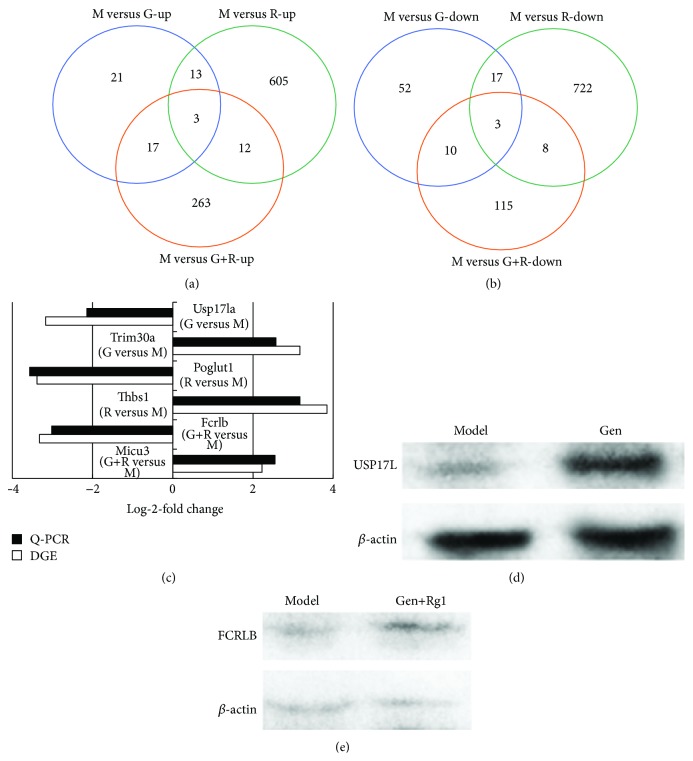
Venn diagrams show comparative analysis of gene expression profiles among geniposide-, ginsenoside-Rg1-, and the combination-treated groups. (a) Upregulated differentially expressed genes overlapping and nonoverlapping among the 3 treatment groups. (b) Downregulated differentially expressed genes overlapping and nonoverlapping among the 3 treatment groups. (c) Real-time qRT-PCR validation of the DGE results. DGE results compared to qRT-PCR results relative to the Ct value. The most clearly upregulated or downregulated genes in the 3 treatment groups from DGE data (FDR *p* < 0.05; fold change >1.5) were selected. *β*-actin was used as a normalizer for each experiment. Expression changes are depicted as log-2-fold change (*x*-axis). Gene symbols are shown on the other side of the column. (d) USP17L protein expression in model group and geniposide-treatment group determined by western blotting. (e) Fcrlb protein expression in model group and combination-treated group determined by western blotting. M versus G-up (down), up- (down-) regulated genes in geniposide-treated group compared with model group; M versus R-up (down), up- (down-) regulated genes in ginsenoside-Rg1-treated group compared with model group; M versus G+R-up (down), up- (down-) regulated genes in combination-treated group compared with model group. G versus M, differential expressed genes in geniposide-treated group compared with model group; R versus M, differential expressed genes in ginsenoside-Rg1-treated group compared with model group; G+R versus M, differential expressed genes in combination-treated group compared with model group.

Figure 3(a) displayed the upregulated differentially expressed genes overlapping and nonoverlapping among the 3 different treated groups, which showed that (1) 3 upregulated differentially expressed genes overlapped among the 3 groups; (2) 17 upregulated differentially expressed genes overlapped between the geniposide monotherapy and combinated treatment, 12 upregulated differentially expressed genes overlapped between the ginsenoside Rg1 monotherapy and combinated treatment, and 13 upregulated differentially expressed genes overlapped between the geniposide and ginsenoside Rg1 monotherapy groups; and (3) 21, 605, or 263 differentially expressed genes were only upregulated in the geniposide monotherapy, ginsenoside Rg1 monotherapy, or combination-treated groups, respectively.

Figure 3(b) showed the downregulated differentially expressed genes overlapping and nonoverlapping among the 3 treatment groups, which demonstrated that (1) 3 downregulated differentially expressed genes overlapped among the 3 groups; (2) 10 downregulated differentially expressed genes overlapped between the geniposide monotherapy and combination-treated groups, 8 upregulated differentially expressed genes overlapped between the ginsenoside Rg1 monotherapy and combination-treated groups, and 17 downregulated differentially expressed genes overlapped between the geniposide and ginsenoside Rg1 monotherapy groups; and (3) 52, 722, or 115 differentially expressed genes were only downregulated in the geniposide-, ginsenoside-Rg1-, and combination-treated groups, respectively.

### 3.4. RT-PCR and Western Blot Validation

To identify whether sequencing-based gene expression detection was reliable for determination of gene expression patterns, we performed SYBR-Green-based real-time qRT-PCR on the most obvious upregulated or downregulated genes in the 3 treatment groups from Digital Gene Expression Profiling (DGE) data (FDR *p* < 0.05; fold change >1.5). All the expressions of the selected genes were in agreement with the sequencing analyses in terms of the direction of the observed differential expression (Figure 3(c)). Specifically, the observed log-2-fold changes with qRT-PCR of the most significant upregulated genes (Usp17la, Poglut1, and Fcrlb in three groups) had the same positive tendency as obtained with the DGE data. Equally, the observed log-2-fold changes with qRT-PCR of the most significant downregulated genes (Trim30a, Thbs1, and Micu3 in three groups) had the same negative tendency as obtained in the DGE data. In addition, we also validated the upregulated genes of Usp17la in geniposide-treatment group and Fcrlb in combination-treated group at protein level by western blot. As shown in Figures 3(d) and 3(e), the expressions of Usp17la in geniposide-treatment group and Fcrlb in combination-treated group were remarkably upregulated as compared with model group, which displayed the same change trend with the results of RT-PCR.

### 3.5. KEGG Database Analysis

**Figure 4:**
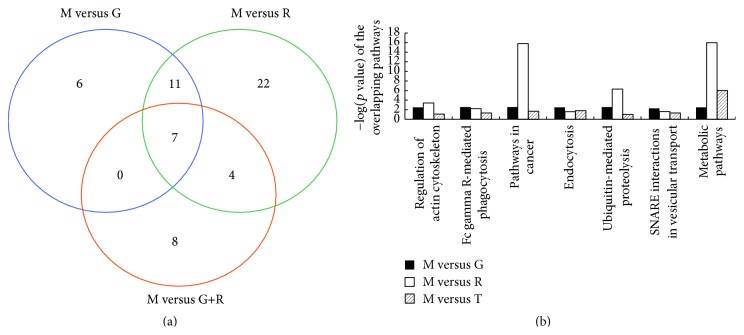
Results of KEGG pathway analysis. (a) Venn diagram of the significant pathways distribution of the 3 treatment groups. (b) Seven overlapping pathways all activated in the 3 treatment groups. G versus M, pathways from differential expressed genes in geniposide-treated group compared with model group; R versus M, pathways from differential expressed genes in ginsenoside-Rg1-treated group compared with model group; G+R versus M, pathways from differential expressed genes in combination-treated group compared with model group.

**Figure 5:**
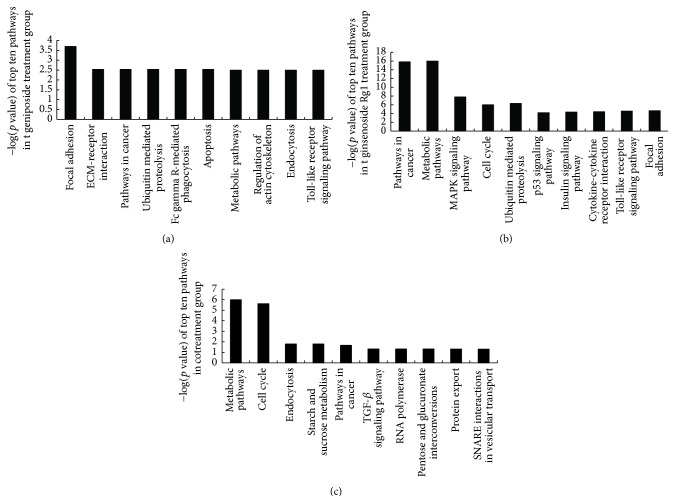
The top 10 pathways activated in the geniposide-treated group (a), ginsenoside-Rg1-treated group (b), and combination-treated group (c).

Based on the KEGG database, 7 overlapping pathways were identified among the 3 drug-treated groups, including regulation of actin cytoskeleton, Fc*γ*R-mediated phagocytosis, and SNARE interactions in vesicular transport (Figures 4(a) and 4(b)). 11 overlapping pathways were noted between the geniposide monotherapy and ginsenoside Rg1 monotherapy comparisons and 4 overlapping pathways were noted between the combinated treatment and ginsenoside Rg1 monotherapy comparisons, which included the TGF-*β* signaling pathway (Figure 5(c)). There was no overlapping pathway between the combinated treatment and geniposide monotherapy comparisons. 6 pathways were markedly activated in the geniposide monotherapy group, and 8 pathways were significantly activated in the ginsenoside Rg1 monotherapy and combined-treatment groups, respectively.

**Figure 6:**
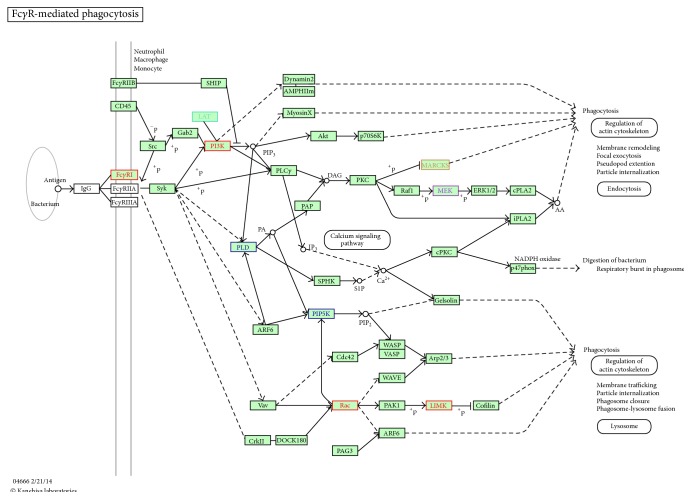
Fc*γ*R-mediated phagocytosis pathway in the KEGG database is the putative pathway which is related to TNF-*α* and TGF-*β*1 expression in microglial cells. The green box represents the altered genes of the Fc*γ*R-mediated phagocytosis pathway in the geniposide-treated group. The red boxes represent the altered genes of the Fc*γ*R-mediated phagocytosis pathway in the ginsenoside-Rg1-treated group. The blue boxes represent the altered genes of the Fc*γ*R-mediated phagocytosis pathway in the combination-treated group. The purple box represents the overlapping altered genes of the Fc*γ*R-mediated phagocytosis pathway in the geniposide- and ginsenoside-Rg1-treated groups. The orange box represents the overlapping altered genes of the Fc*γ*R-mediated phagocytosis pathway among the 3 drug groups.

According to the adjusted *p* value and the relation to microglial cell function, the pathways in the geniposide-treatment group (Figure 5(a)) mainly involved focal adhesion, extracellular matrix-receptor interaction, and ubiquitin-mediated proteolysis. The related pathways of the ginsenoside Rg1-treatment group (Figure 5(b)) mainly involved the cell cycle, mitogen-activated protein kinase (MAPK) signaling pathway, and ubiquitin-mediated proteolysis. The related pathways of the combination-treated group (Figure 5(c)) mainly focused on metabolic pathways, cell cycle, and TGF-*β* signaling pathway. According to the pathological role of microglia in ischemic stroke, we expected that the Fc*γ*R-mediated phagocytosis pathway (Figure 6) plays an essential role in microglial function, which was activated in the overlapping pathways identified among the 3 drug-treated groups.

### 3.6. Cluster Analysis

**Figure 7:**
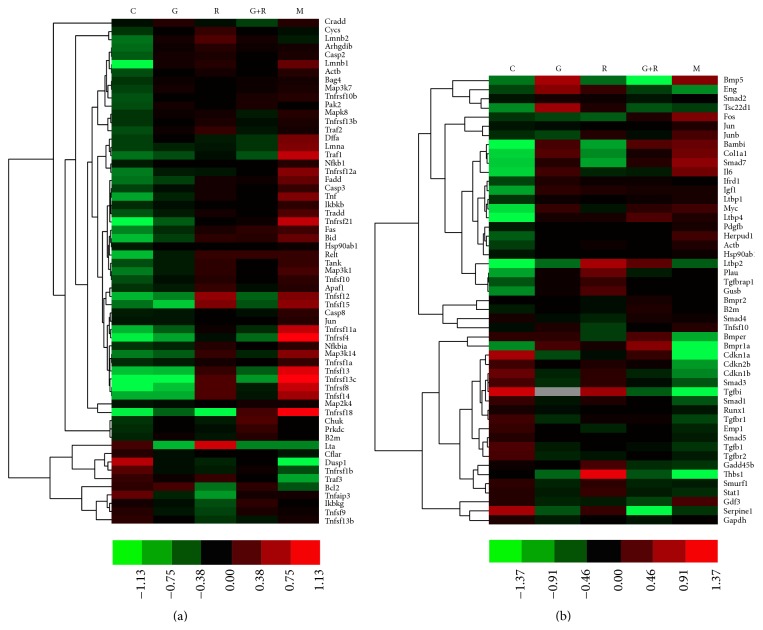
(a) Cluster analysis shows that most of the genes in the TNF-*α* pathway in the control and geniposide-treated groups were clustered, which indicated that the expression pattern of the 2 groups was similar to a certain extent. (b) Cluster analysis demonstrates that the expression pattern of the ginsenoside-Rg1-treated group was more similar to that of the control group. Some of the genes included in TNF-*α* and TGF-*β* pathway are listed on the right.

**Figure 8:**
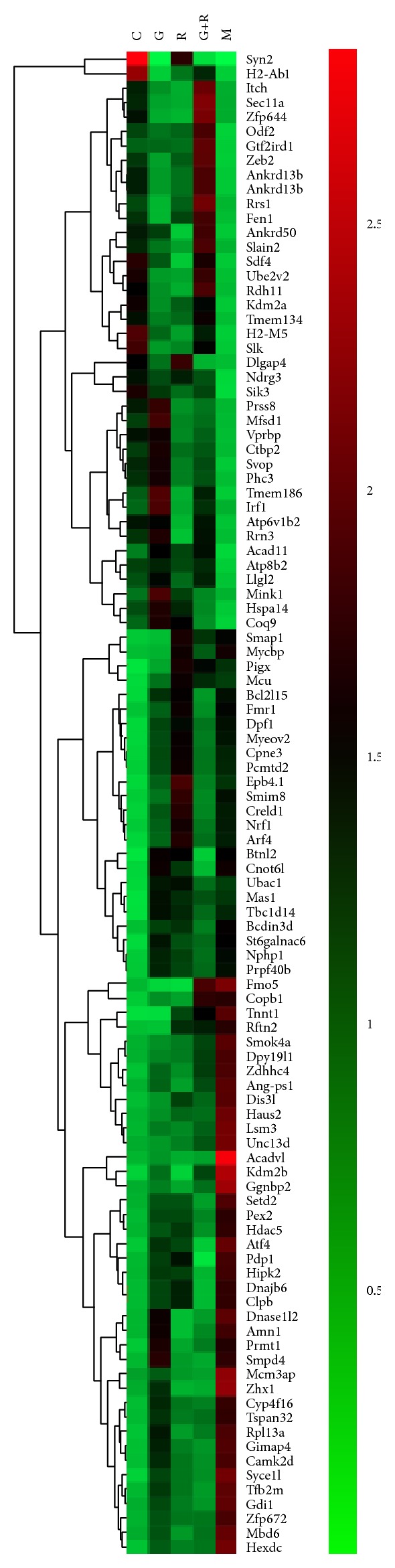
Cluster analysis demonstrates that the genes in the model group and the control group were classified into two categories. The genes cluster of combination-treated group was most nearest to the control group.

To understand the synergistic effect of geniposide and ginsenoside Rg1 on TNF-*α* and TGF-*β* expression pattern in ischemia-activated microglia, we clarified the distribution of genes in TNF-*α* and TGF-*β* pathways by cluster analysis.  Figure 7(a) indicated that genes in the control and model groups were more clustered into 2 different categories in TNF-*α* pathway, most of which could activate nuclear factor- (NF-) *κ*B or induce cell apoptosis. Most of the genes in the TNF-*α* pathway in the control and geniposide individual group were clustered, which indicated that the expression patterns of the 2 groups were similar to a certain extent.  Figure 7(b) demonstrated that the genes in the control and model groups were also clustered into 2 different categories, most of which could inhibit the microglial cell cycle. In the TGF-*β* pathway, the expression pattern of the ginsenoside-Rg1-treated group was more similar to that of the control group. In addition, results from a thorough cluster analysis (Figure 8) displayed that the genes in the model group and the control group were classified into two categories. Certain genes in geniposide-treated group and ginsenoside Rg1 group were clustered together, suggesting the similar targets of the two compounds. The genes cluster of combination-treated group was most nearest to the control group which indicated that the changed genes after being treated by combination were similar to the genes of normal cultured cells.

## 4. Discussion

Geniposide is an iridoid glycoside isolated from Gardenia and possesses diverse pharmacological activities such as antioxidative [21], antitumor [22], antiasthma, and antidiabetic [23, 24] effects. Ginsenoside Rg1 is one of the most active and abundant steroid saponins, with antiapoptotic [25] and neuroprotective [26] effects. Stroke is more complex than initially anticipated because it is often caused by multiple molecular abnormalities, rather than being the result of a single effect [27]. Consequently, multicomponent treatments interact with multiple targets simultaneously and are considered as a rational and efficient form of therapy for complex diseases [28, 29]. We and other researchers have already demonstrated that the prescription (Tongluo Jiunao injection) combined with ginsenoside and ginsenoside Rg1 is effective for treatment of stroke via their anti-inflammatory, neuroprotective, and neurotrophic roles [30, 31]. In this study, we demonstrated for the first time a potent synergistic effect of ginsenoside and ginsenoside Rg1 on OGD-injured microglia. The combined results indicate that ginsenoside Rg1 monotherapy suppressed NO release, which might have been achieved through inhibition of cell viability. In comparison, geniposide monotherapy and combinated treatment both increase cell viability and reduce NO content simultaneously. The effect of combinated treatment on cell viability improvement was prior to that of ginsenoside monotherapy, which showed synergistic effect of compatibility. We propose that the effects of geniposide and ginsenoside Rg1 on microglia have their own emphases.

Previous studies have pointed out that microglia possess dual functions (i.e., neuroprotection/neurotrophy and neuron destruction/neurotoxicity) because microglia produce cytotoxic proinflammatory factors that induce neuronal cell death, as well as neurotrophic factors that support neuronal survival. Therefore, from the balance of proinflammatory cytokine TNF-*α* and anti-inflammatory cytokine TGF-*β*1 secreted by microglial cells, we wanted to understand further the synergistic effect of ginsenoside and ginsenoside Rg1 for the treatment of OGD-injured microglia. From the consistent results of microglial cell secretion and expression, we found that geniposide monotherapy inhibited TNF-*α* but not TGF-*β*1 expression. In contrast, a ginsenoside Rg1 monotherapy inhibited TGF-*β*1 but not TNF-*α* expression. However, the combinated treatment had a synergic effect on expression of TNF-*α* and TGF-*β*1, because combinated treatment could both reduce TNF-*α* level and improve TGF-*β*1 level which indicated a balance between proinflammatory cytokine and anti-inflammatory cytokine.

The most significant upregulated gene was Trim30a in the geniposide-treatment group, which negatively regulated Toll-like-receptor-mediated NF-*κ*B activation by targeting degradation of TGF*β*-activated kinase 1- (TAK1-) binding proteins 2 and 3 (TAB2 and TAB3) [32]. Therefore, upregulation of Trim30a by geniposide partly accounts for the suppression of TNF-*α* level. The most significantly downregulated gene in the geniposide-treatment group was Usp17la. USP17 depletion significantly impairs G1-S transition and blocks cell proliferation [33], which indicate that downregulation of Usp17la by geniposide could inhibit the cell cycle and therefore microglial cell proliferation. The most upregulated gene in ginsenoside Rg1-treatment group was Thbs1 (thrombospondin 1), an extracellular matrix molecule, which is involved in activation of TGF-*β* [34] and anti-inflammatory activity [35]. The most downregulated gene in the ginsenoside Rg1-treatment group was Poglut1 (protein O-glucosyltransferase), which enhances Notch signaling activation [36]. Consequently, downregulation of Poglut1 by ginsenoside Rg1 resulted in Notch inhibition. When Notch1 transcription in microglia is inhibited, upregulation of the expression of proinflammatory cytokines is observed [37]. Combining the most clearly upregulated and downregulated genes in the ginsenoside-Rg1-treated group, we think that the improvement in TGF-*β*1 may be due to upregulated Thbs1, and failure of TNF-*α* inhibition of ginsenoside Rg1 may be due to the downregulated gene Poglut1. The most clearly downregulated gene in combinated group was Fcrlb, which might retain secreted IgG in cells [38]. In considering the Fcrlb function, we reasoned that less Fcrlb might secrete less immunoglobulin. Emerging evidence shows that congeneric IgG stimulation might lead to proinflammatory cytokine production, probably via a myeloid differentiation factor 88-dependent pathway [39]. Combinated treatment could suppress proinflammatory cytokine secretion on the premise of cellular energy metabolism.

The genes in the TNF-*α* and TGF-*β* pathway cluster analysis showed that most of the genes in the TNF-*α* pathway in the geniposide-treated group were clustered with those in the control group, which indicated that the expression pattern of the two groups was similar. In the TGF-*β* pathway, the expression pattern in the ginsenoside-Rg1-treated group was more similar to that in the control group. This result is consistent with that of TNF-*α* and TGF-*β* expression, which indicates that geniposide has a greater effect on the TNF-*α* pathway in microglial cells. In contrast, ginsenoside Rg1 have a greater effect on the TGF-*β* pathway.

According to the KEGG analysis, the combinated treatment-ginsenoside Rg1 monotherapy comparisons had an overlapping TGF-*β* signaling pathway, which is consistent with the results of ELISA, western blotting, and TGF-*β* pathway cluster analysis. This indicates that effect of ginsenoside Rg1 on microglial is focused more on the regulation of TGF-*β*. Extracellular matrix-receptor interaction, as one of pathways in geniposide-treatment group, is involved in the decreasing the activity of matrix metalloproteinase-2 [40] and the TNF-*α*-mediated matrix metalloproteinase-1 and matrix metalloproteinase-3 by geniposide [41]. MAPK pathway was identified as one of pathways in ginsenoside Rg1-treatment group. It was reported that ginsenoside Rg1 significantly attenuates overactivation of microglial cells by repressing expression levels extracellular signal-regulated kinase 1/2 (ERK1/2), c-Jun N-terminal protein kinase (JNK), and p38 mitogen-activated protein kinase (p38 MAPK) [42]. Fc*γ*R-mediated phagocytosis is the overlapping pathway for the 3 drug treatments and is related to microglial cell function. Microglia become capable phagocytic cells through interaction with their cognate Fc*γ*R [43] during transformation from a surveillance state to an activated phenotype in response to brain injury. Cerebral ischemia injury involves low-level chronic inflammation, and studies have shown that Fc*γ*R could induce TNF-*α* mRNA expression and MIP-1*α* production [44, 45]. There is evidence that Fc*γ*R might help to produce anti-inflammatory cytokines such as IL-10 and TGF-*β* that play a role in susceptibility to infection [46]. Consequently, Fc*γ*R plays a key role in both TNF-*α* and TGF-*β*1. Based on our data, we think that independent use of either geniposide or ginsenoside Rg1 could make Fc*γ*R exert a different regulated effect on TNF-*α* or TGF-*β*1. Geniposide monotherapy induced Fc*γ*R to exert an effect on TNF-*α*. In contrast, ginsenoside Rg1 monotherapy induced Fc*γ*R to exert an effect on TGF-*β*1. Combinated treatment affects Fc*γ*R, which plays an important role in microglial cell function through the balance of TNF-*α* and TGF-*β*1. The synergistic effects of geniposide and ginsenoside Rg1 are linked to their pharmacological effect through the Fc*γ*R-mediated phagocytosis pathway, which differs from the independent use of either geniposide or ginsenoside Rg1.

In conclusion, our findings indicate that geniposide monotherapy or ginsenoside monotherapy exerts a different regulatory effect on OGD-induced microglial cells. Combined use of geniposide and ginsenoside Rg1 has a synergistic effect, which is mainly characterized by the balance of proinflammatory cytokine TNF-*α* and anti-inflammatory cytokine TGF-*β*1. This synergistic effect may correlate with the most clearly changed genes and the Fc*γ*R-mediated phagocytosis pathway. Nevertheless, further studies are needed to validate the mechanism of the synergistic effect associated with microglial cells.

## Supplementary Material

The chemical structure of geniposide is C17H24O10, and its molecular weight is 404.36. The chemical structure of ginsenoside Rg1 is C42H72O14 and its molecular weight is 801.01.
