# Supplementary material for: Synergistic Use of Geniposide and Ginsenoside Rg1 Balance Microglial TNF-α and TGF-β1 following Oxygen-Glucose Deprivation In Vitro: A Genome-Wide Survey
Source: Evid Based Complement Alternat Med. 2015 Nov 29;2015:756346. doi: 10.1155/2015/756346 (PMC4677035; doi:10.1155/2015/756346)
Supplement: Supplementary file 1 — The chemical structure of geniposide is C17H24O10, and its molecular weight is 404.36. The chemical structure of ginsenoside Rg1 is C42H72O14 and its molecular weight is 801.01. [file 756346.f1.doc]

(A)


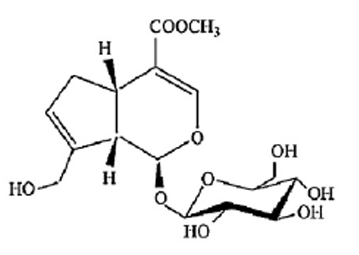


(B)


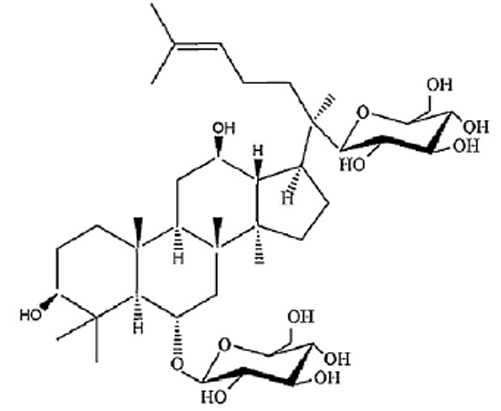


Figure legend

The chemical structure of (A) geniposide (C_17_H_24_O_10_, molecular weight: 404.36) and (B) ginsenoside Rg1 (C_42_H_72_O_14_, molecular weight: 801.01).
